# Author Correction: tgCRISPRi: efficient gene knock-down using truncated gRNAs and catalytically active Cas9

**DOI:** 10.1038/s41467-024-47644-3

**Published:** 2024-04-12

**Authors:** Ankush Auradkar, Annabel Guichard, Saluja Kaduwal, Marketta Sneider, Ethan Bier

**Affiliations:** 1 Department of Cell and Developmental Biology, University of California, San Diego, 9500 Gilman Drive, La Jolla, CA 92093-0335 USA; 2 Tata Institute for Genetics and Society - UCSD, La Jolla, USA

**Keywords:** Gene regulation, CRISPR-Cas systems, Genetic techniques

Correction to: *Nature Communications* 10.1038/s41467-023-40836-3, published online 11 September 2023

The original version of the Supplementary Information associated with this Article contained an error in Supplementary Table 1. The HTML has been updated to include a corrected version of the Supplementary Information.

### Supplementary information

Supplementary Information

