# Supplementary material for: tgCRISPRi: efficient gene knock-down using truncated gRNAs and catalytically active Cas9
Source: Nat Commun. 2023 Sep 11;14:5587. doi: 10.1038/s41467-023-40836-3 (PMC10495392; doi:10.1038/s41467-023-40836-3)
Supplement: Supplementary file 1 — Supplementary Information [file 41467_2023_40836_MOESM1_ESM.pdf]

tgCRISPRi: Efficient gene knock-down using truncated gRNAs and catalytically active Cas9

Ankush Auradkar[1], Annabel Guichard[1], Saluja Kaduwal[1], Marketta Sneider[1], and Ethan Bier[1,2] *

[1]Department of Cell and Developmental Biology, University of California, San Diego, 9500 Gilman Drive, La Jolla, CA 92093-0335

[2]Tata Institute for Genetics and Society - UCSD

* Corresponding author

E-mail: ebier@ucsd.edu

This Supplementary Information file contains:

1. **Main Supplementary Figures** (Sup. Figure 1-7)
2. **Main Supplementary Tables** (Sup. Table 1-2)

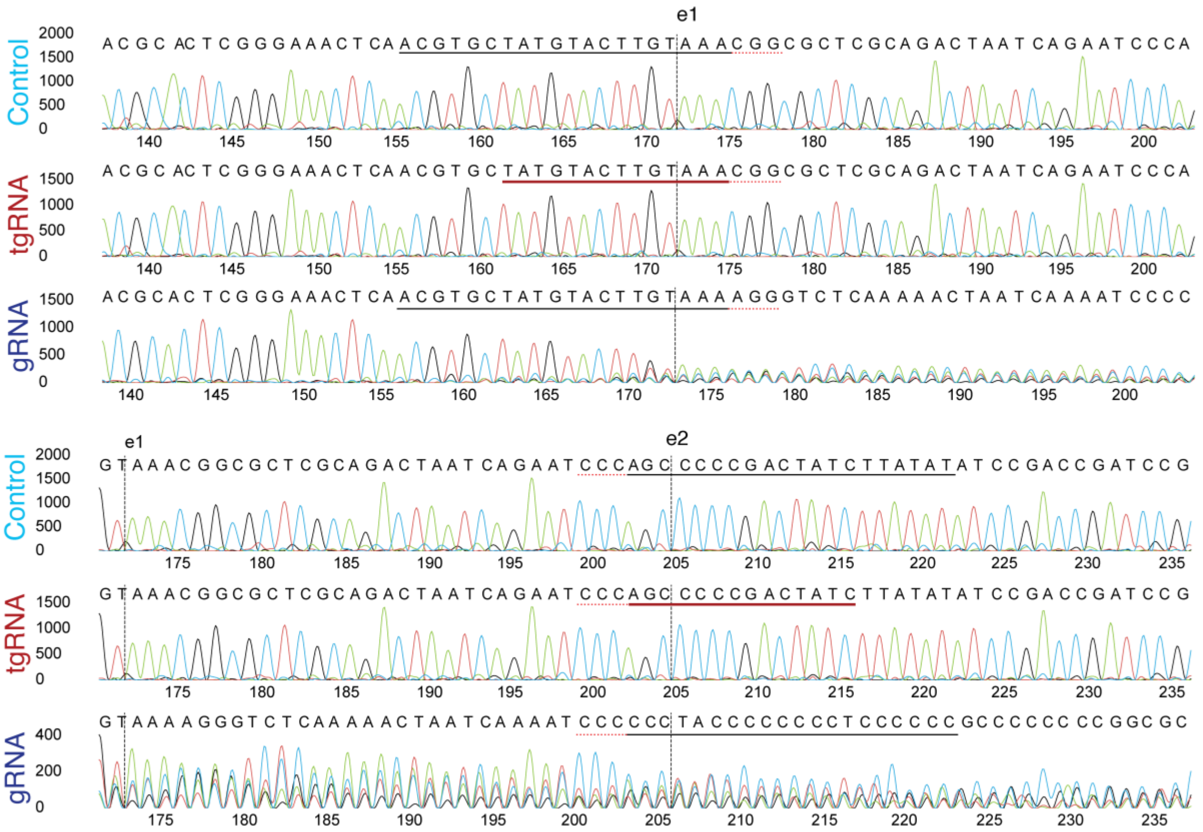

Sup. Figure 1. Comparing CRISPR and tgCRISPRi DNA sequence chromatograms to reveal NHEJ events for the e gene. Control e+ DNA sequence around the tg/gRNA-e2 and tg/gRNA-e1 cut site is shown on top of the 1st and 4th row, respectively. Chromatograms for control (+/vasa-Cas9) animals (1st and 4th row) show no NHEJ or double peaks. Upon introduction of tgRNA-e2 and e1, peaks appear similar to the control (2nd and 5th row). In gRNA-e2 and e1 expressing animals (3rd and 6th row), multiple peaks appear around the cut site, confirming DSB and DNA editing.

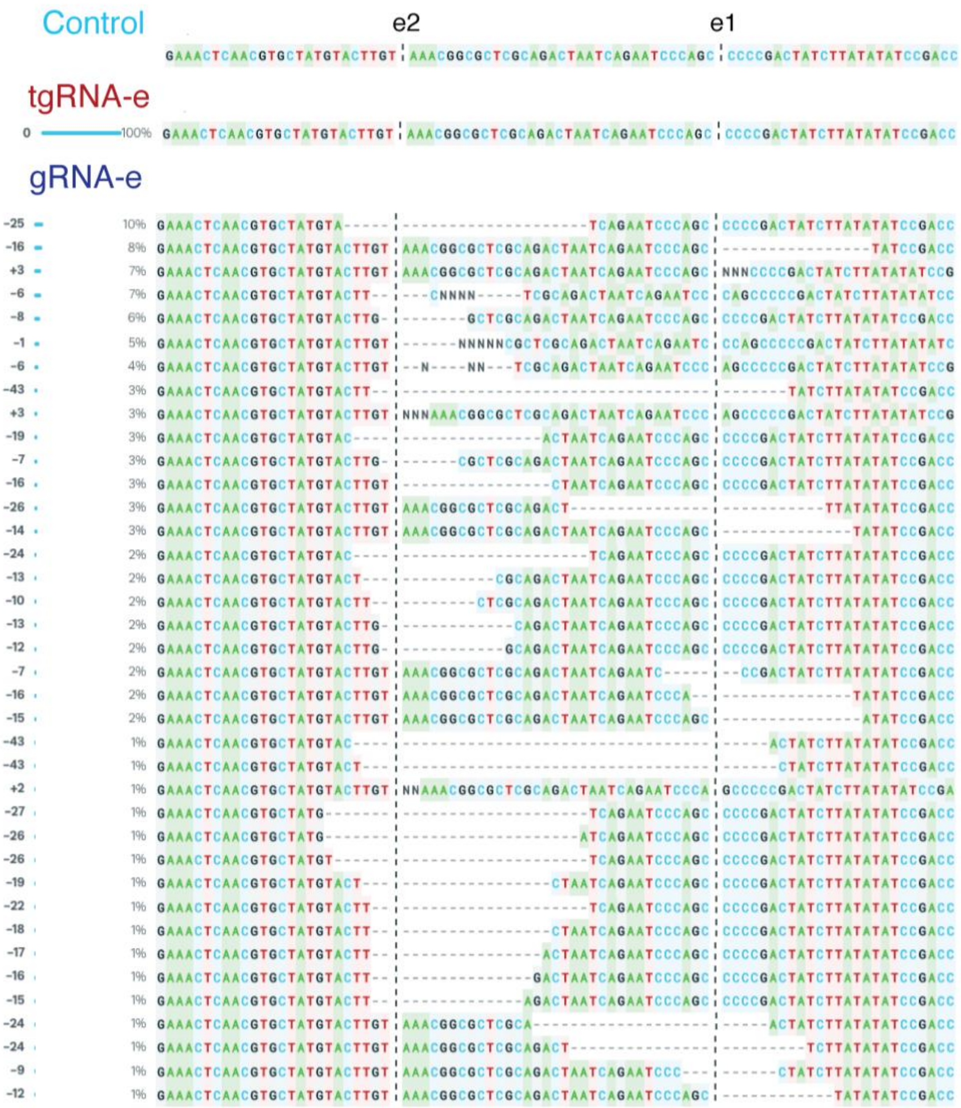

Sup. Figure 2. Detection of tgCRISPRi and CRISPR-induced mutations for guide targeting e gene locus. Percentage of indels as shown by Synthego ICE online tool. F_1 progeny from control, tgRNAs, and gRNAs master flies were sequenced. In control and tgRNAs F_1 progeny, 100% of alleles were wildtype. In gRNAs F_1 progeny, 100% of alleles exhibited NHEJ events. Sequences on the right side show the mutation rates with the sizes of deletions (−) and insertions (+), with the percentage of indels and KO score for each allele.

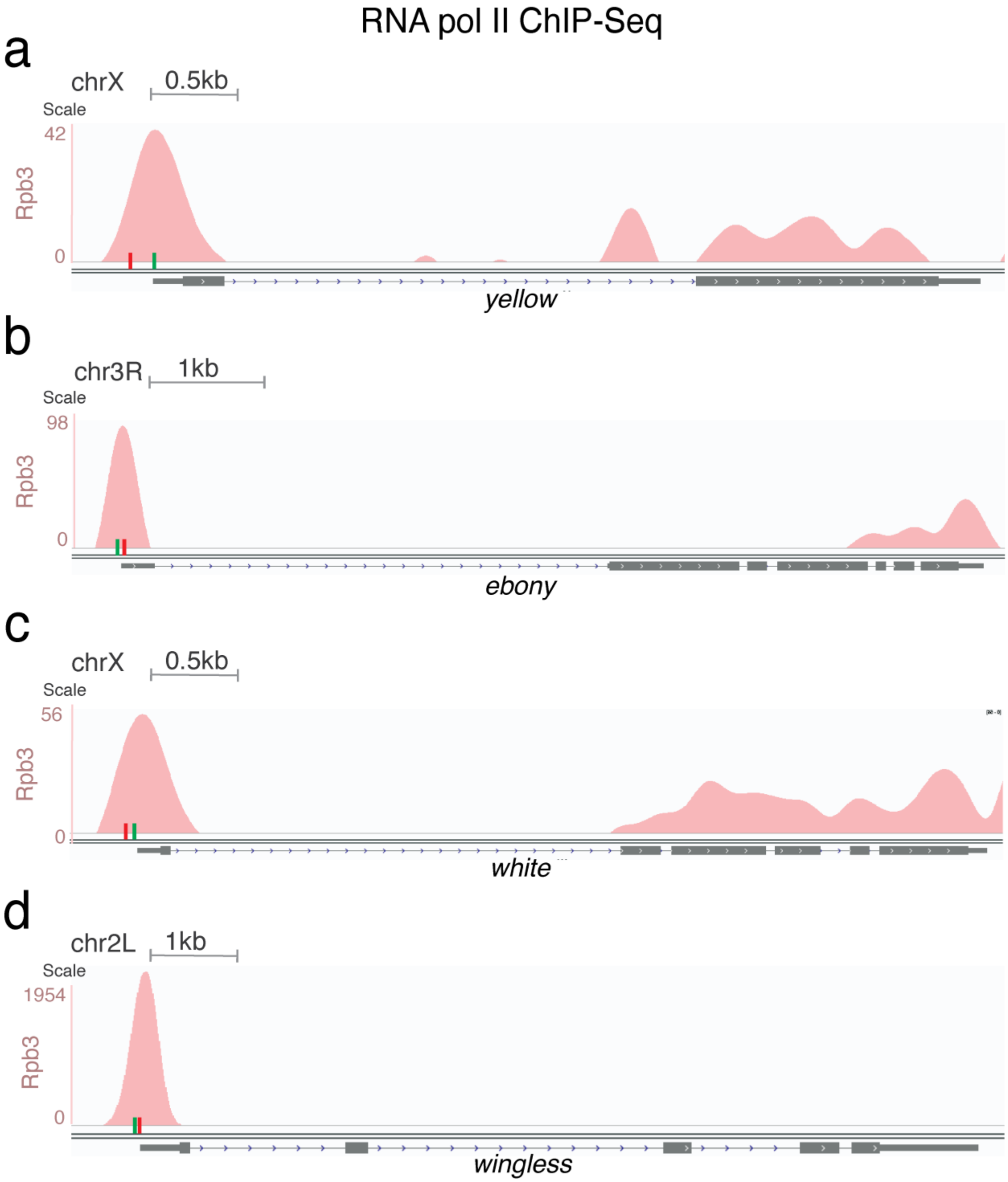

Sup. Figure 3. (a-d) Genome browser view of RNA polymerase II subunit C (Rpb3) at *yellow*, *ebony*, *white, and wingless* genes, highlighted with locations of successful tgRNAs (green bar) and inactive tgRNA (red bar) target sites relative to the TSS.

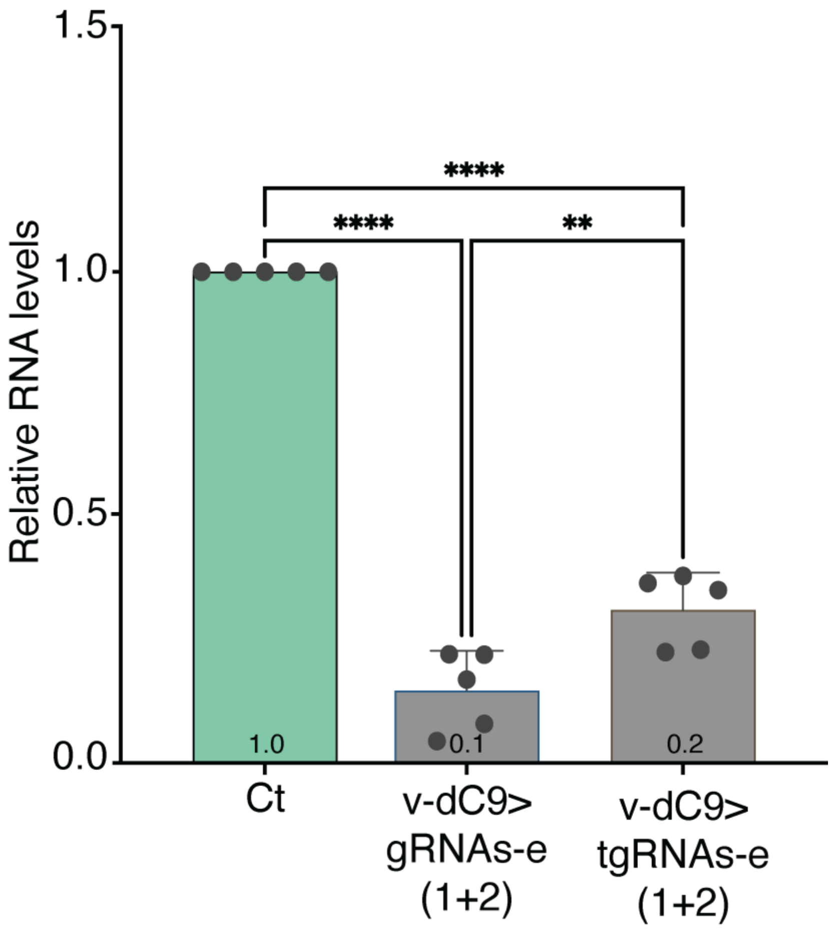

Sup. Figure 4. Graph shows the comparison of relative RNA level using RT-qPCR of e gene transcript from control (Ct = +/vasa-dCas9), gRNAs-e(1+2)/vasa-dCas9, tgRNAs-e(1+2)/vasa-dCas9 adult flies. Transcript levels of *rp49* were used as a reference. Error bars represent standard error of the mean of five independent experiments and analyzed using One-way ANOVA. Data plotted as mean ± SD, $^{**}p=0.0044$, $^{****}p < 0.0001$.

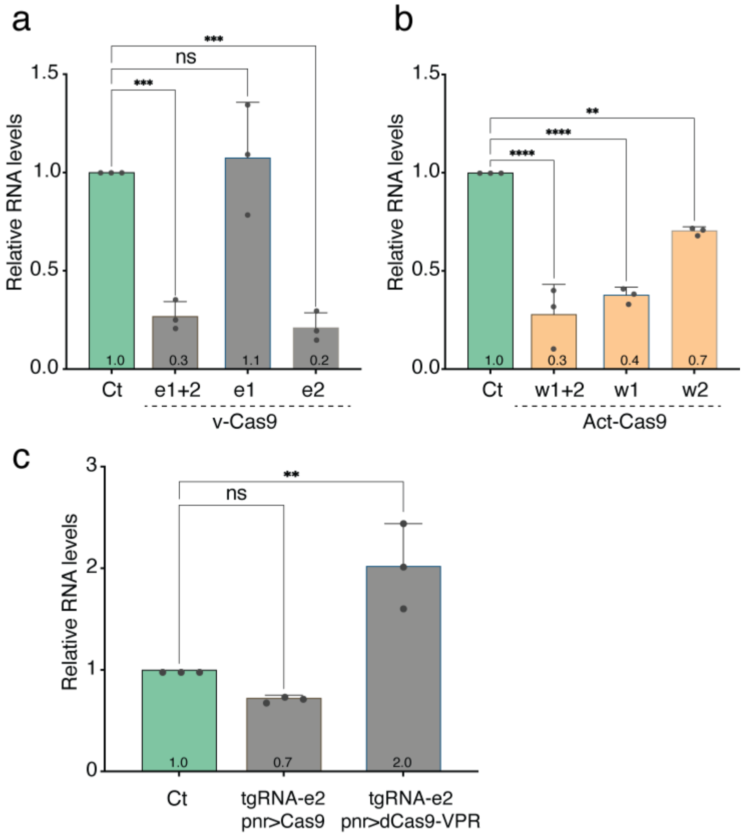

Sup. Figure 5. (a) Graph shows the comparison of relative RNA level using RT-qPCR of e gene transcript from control (Ct = +/vasa-Cas9), tgRNA-e(1+2)/vasa-Cas9 (***p-value= 0.0009), tgRNA-e1/vasa-Cas9 (ns p-value= 0.8), and tgRNA-e2/vasa-Cas9 (***p-value= 0.0005) adult flies. **(b)** Graph shows the comparison of relative RNA level using RT-qPCR of *w* gene transcript from control (Ct = +/Act-Cas9), tgRNA-w(1+2)/Act-Cas9 (****p-value< 0.0001), tgRNA-w1/Act-Cas9 (****p-value< 0.0001) and tgRNA-w2/Act-Cas9 (**p-value= 0.0049) adult flies. **(c)** Graph shows the comparison of relative RNA level using RT-qPCR of e gene transcript from control (Ct = +/pnr>Cas9), tgRNA-e2/pnr>Cas9 (repression) (ns p-value= 0.3) and tgRNA-e2/pnr>dCas9-VPR (activation) (**p-value= 0.0033) adult flies. Transcript levels of *rp49* were used as a reference. Error bars represent standard deviation of the mean of three independent experiments and analyzed using One-way ANOVA. Data plotted as mean ± SD.

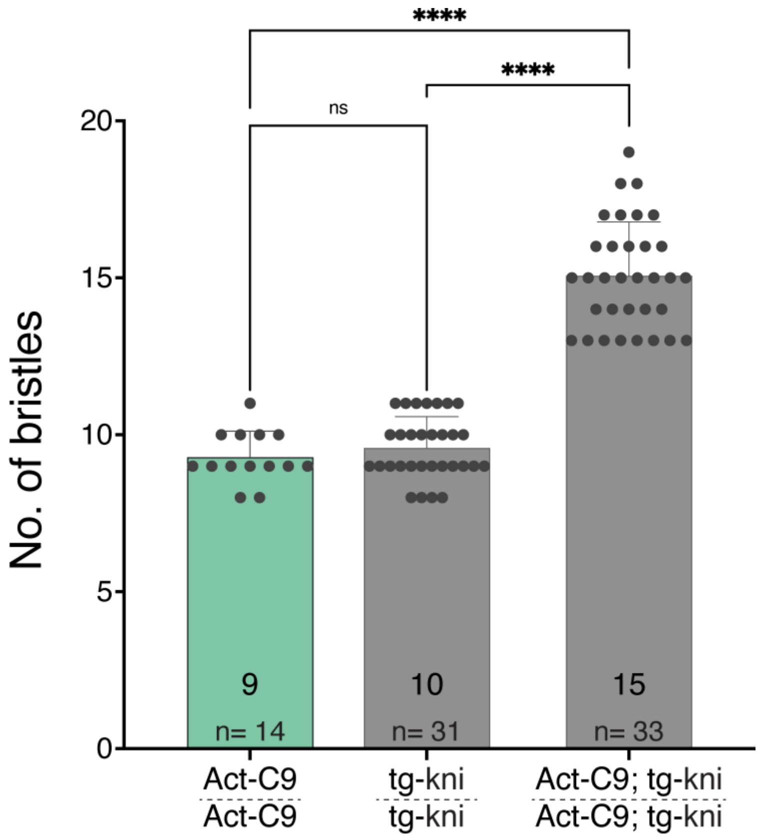

Sup. Figure 6. Graph shows the comparison of number of bristles after the merging of the L2 vein with the wing margin in control flies homozygous for *Act-Cas9* and *tgRNAs-kni(1+2)*, with flies homozygous for *Act-Cas9; tgRNAs-kni(1+2)* flies. Data were analyzed using One-way ANOVA. Data plotted as mean ± SD ****$p < 0.0001$, ns not significant. n = number of samples.

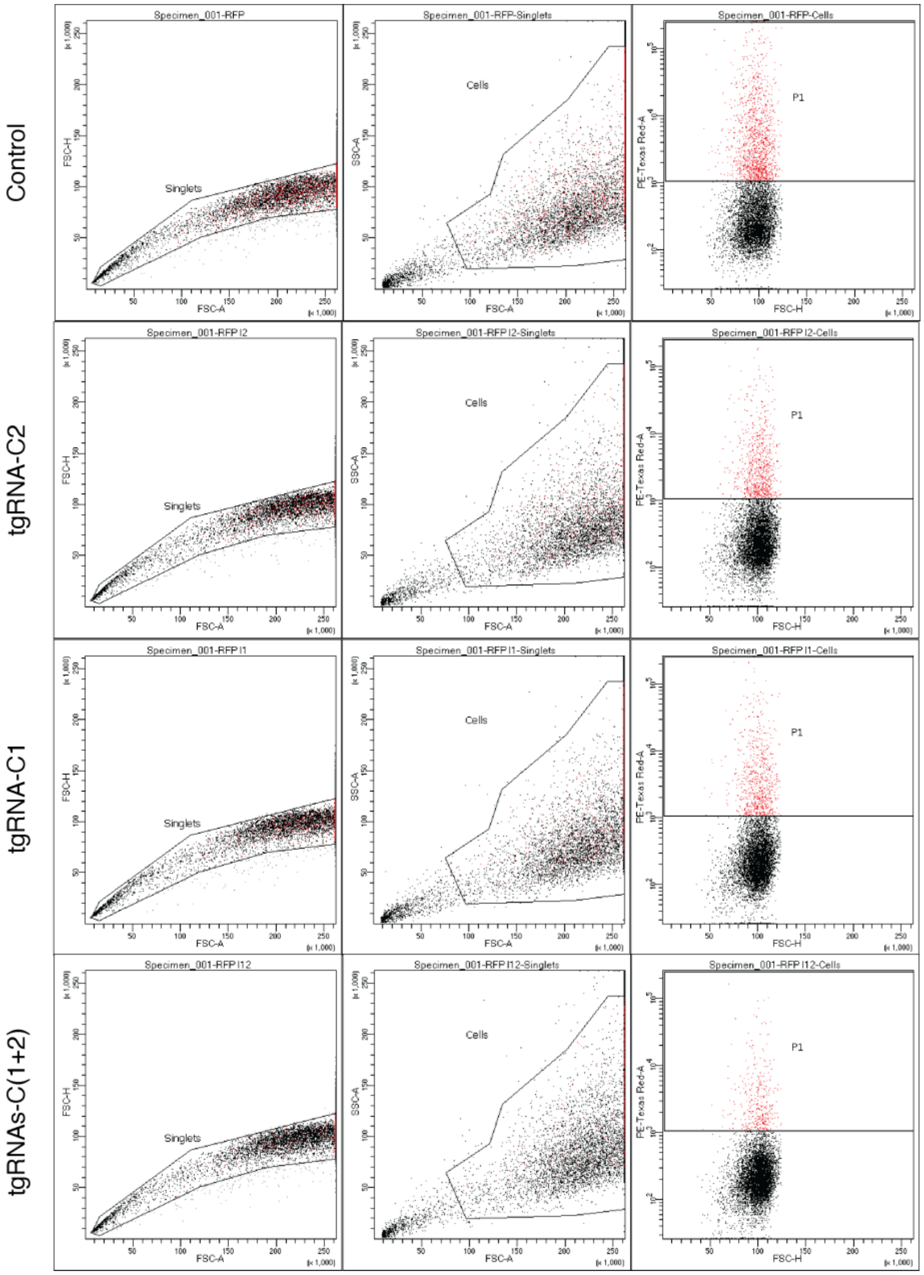

Sup. Figure 7. Gating strategy of FACS sorting for analyses of mCherry fluorescence in HEK293 cells transfected with CMV-mCherry plus Cas9-tgRNA-ve plasmid (control) or CMV-mCherry plus Cas9-tgRNA-c2 plasmid (tgRNA-c2), or CMV-mCherry plus Cas9-tgRNA-c1 plasmid (tgRNA-c1), or CMV-mCherry plus Cas9-tgRNAs-c(1+2) plasmid (tgRNAs-c(1+2)). mCherry-positive cells are shown in red.

Supplementary Table 1: tg/gRNA sequences

tg/gRNA	Sequences
Y2_full	GATTCGGGTGGTTCAGTGTTC
Y1_full	GCGTGGCGCGGTAACGACTGG
E2_full	GATATAAGATAGTCGGGGGCT
E1_full	GACGTGCTATGTACTTGTAAA
Y2	GTGGTTCAGTGTTC
Y1	GCGGTAACGACTGG
E2	GATAGTCGGGGGCT
E1	GTATGTACTTGTAAA
Wg2	GACAAACGCAGAGT
Wg1	GTTTCAGTTAAGCGT
W2	GTAACGCTACAAA
W1	GACGAAGCTCCAAG
kni1	GCCGCCCTTCAGACC
kni2	GGGCATAATTAGGG
Scr1	GATTAACAAGAATGG
Scr2	GAGCTGGAGAGAGG
CMV2	GCGTGTACGGTGGG
CMV1	GCTAGCGGATCTGA

Supplementary Table 2: Primers

657y	tatttctagctctaaaacccagtcgttaccgcgacgttaaattgaaaataggtc
658y	atatccgggtgaacttcgtggttcagtgttcgttttagagctagaaatagcaag
659e	tatttctagctctaaaactttacaagtacatacgacgttaaattgaaaataggtc
660e	atatccgggtgaacttcgatagtcgggggctgttttagagctagaaatagcaag
700_y1	aaacgaacactgaaccac
701_y1	gtcgtggttcagtgttc
702_y2	gtcgcggtaacgactgg
703_y2	aaacccagtcgttaccg
704_e1	gtcgatagtcgggggct
705_e1	aaacagcccccgactat
706_e2	gtcgtatgtacttgtaaa
707_e2	aaactttacaagtacata
711_yfull	atatccgggtgaacttcgattcgggtggttcagtgttcgttttagagctagaaatagcaag
712_yfull	tatttctagctctaaaacccagtcgttaccgcgccacgcgacgttaaattgaaaataggtc
713_efull	atatccgggtgaacttcgatataagatagtcgggggctgttttagagctagaaatagcaag
714_efull	tatttctagctctaaaactttacaagtacatagcacgtcgacgttaaattgaaaataggtc
779_wg	atatccgggtgaacttcgacaaacgcagagtgttttagagctagaaatagcaag
780_wg	tatttctagctctaaaacacgcttaactgaaacgacgttaaattgaaaataggtc
783_w	atatccgggtgaacttcgtaacgctacaaagttttagagctagaaatagcaag
784_w	tatttctagctctaaaaccttggagcttcgtcgacgttaaattgaaaataggtc
807_wg1	atagacctattttcaatttaacgtcgacaaacgcagagtgttttagagctagaaatagcaagtt
808_wg2	atagacctattttctttcgtcgtttcagttgcgtgttttagagctagatagcgtt
809_w1	atagacctattttcaatttaacgtcgtaacgctacaaagttttagagctagaaatagcaagtt
810_w2	atagacctattttcaatttaacgtcgacgaagctccaaggttttagagctagaaatagcaagtt
753_cmv2	caccgctagcggatctga
754_cmv2	aaactcagatccgctagc
755_cmv1	caccgcgtgtacggtggg
756_cmv1	aaccccaccgtacacgc
829_w_ rt-PCR	aggcagcaaacacccatctg
830_w_ rt-PCR	cccgaagccctggttaatg
833_e_rt-PCR	cttagtgtgaaacggccacag
834_e_ rt-PCR	gcagcgaacccatcttgaa
835_y_ rt-PCR	tgtgaccctgatcaccttgg
836_y_ rt-PCR	tcgggtattcgggaaagca
837_rp49	tcggatcgatatgctaagctg
838_rp49	tcgatccgtaaccgatgttg
