# Supplementary material for: tgCRISPRi: efficient gene knock-down using truncated gRNAs and catalytically active Cas9
Source: Nat Commun. 2023 Sep 11;14:5587. doi: 10.1038/s41467-023-40836-3 (PMC10495392; doi:10.1038/s41467-023-40836-3)
Supplement: Supplementary file 2 — Reporting Summary [file 41467_2023_40836_MOESM2_ESM.pdf]

nature portfolio

Corresponding author(s): Ethan Bier

Last updated by author(s): Jul 10, 2023

Reporting Summary

Nature Portfolio wishes to improve the reproducibility of the work that we publish. This form provides structure for consistency and transparency in reporting. For further information on Nature Portfolio policies, see our Editorial Policies and the Editorial Policy Checklist.

Statistics

For all statistical analyses, confirm that the following items are present in the figure legend, table legend, main text, or Methods section.

n/a	Confirmed	
☐	☒	The exact sample size (*n*) for each experimental group/condition, given as a discrete number and unit of measurement
☐	☒	A statement on whether measurements were taken from distinct samples or whether the same sample was measured repeatedly
☐	☒	The statistical test(s) used AND whether they are one- or two-sided<br>*Only common tests should be described solely by name; describe more complex techniques in the Methods section.*
☒	☐	A description of all covariates tested
☒	☐	A description of any assumptions or corrections, such as tests of normality and adjustment for multiple comparisons
☐	☒	A full description of the statistical parameters including central tendency (e.g. means) or other basic estimates (e.g. regression coefficient) AND variation (e.g. standard deviation) or associated estimates of uncertainty (e.g. confidence intervals)
☐	☒	For null hypothesis testing, the test statistic (e.g. *F*, *t*, *r*) with confidence intervals, effect sizes, degrees of freedom and *P* value noted<br>*Give P values as exact values whenever suitable.*
☒	☐	For Bayesian analysis, information on the choice of priors and Markov chain Monte Carlo settings
☒	☐	For hierarchical and complex designs, identification of the appropriate level for tests and full reporting of outcomes
☒	☐	Estimates of effect sizes (e.g. Cohen's *d*, Pearson's *r*), indicating how they were calculated

Our web collection on statistics for biologists contains articles on many of the points above.

Software and code

Policy information about availability of computer code

Data collection	Zen Pro (2012) blue edition for image acquisition and wing vein measurement. ICE tool by Synthego for Sanger sequencing NHEJ analysis.
Data analysis	Helicon Focus software (v8.2.4) was used to stack the images. Bowtie2 software, MACS2 and IGV software(v2.15) were used for ChIP analysis. Graphs were analyzed and generated using Prism 9. FACS data was analyzed using FACSDiva (v9.0). Figure were made on Adobe Illustrator (2023).

For manuscripts utilizing custom algorithms or software that are central to the research but not yet described in published literature, software must be made available to editors and reviewers. We strongly encourage code deposition in a community repository (e.g. GitHub). See the Nature Portfolio guidelines for submitting code & software for further information.

Data

Policy information about availability of data

All manuscripts must include a data availability statement. This statement should provide the following information, where applicable:

- Accession codes, unique identifiers, or web links for publicly available datasets
- A description of any restrictions on data availability
- For clinical datasets or third party data, please ensure that the statement adheres to our policy

The sequence of all gRNAs, tgRNAs and oligoes used in this study are provided with this paper in Supplementary Table 1 and 2. RNA polymerase II subunit C (Rpb3) ChIP-seq data (GSE101557) was used in this study. Source data is provided in this paper as a Supplementary Data file (1).

Research involving human participants, their data, or biological material

Policy information about studies with human participants or human data. See also policy information about sex, gender (identity/presentation), and sexual orientation and race, ethnicity and racism.

Reporting on sex and gender	N/A
Reporting on race, ethnicity, or other socially relevant groupings	N/A
Population characteristics	N/A
Recruitment	N/A
Ethics oversight	N/A

Note that full information on the approval of the study protocol must also be provided in the manuscript.

Field-specific reporting

Please select the one below that is the best fit for your research. If you are not sure, read the appropriate sections before making your selection.

☒ Life sciences ☐ Behavioural & social sciences ☐ Ecological, evolutionary & environmental sciences

For a reference copy of the document with all sections, see nature.com/documents/nr-reporting-summary-flat.pdf

Life sciences study design

All studies must disclose on these points even when the disclosure is negative.

Sample size	This study included all offspring that were produced from the given cross. However, crosses with less than 10 offspring were not included, as the low number could have been caused by contaminated food. In experiments conducted using HEK293T cells, each replicate required a sample size of > 10000 cells per condition.
Data exclusions	No data was excluded in this study
Replication	We performed triplicate crosses ocfall tgRNAs and gRNAs with Cas9, dead-Cas9, Cas9-VPR, and dead-Cas9-VPR, resulting in the generation of over 10 offspring per cross. The replicates yielded consistent and comparable results, indicating that the experimental conditions were reliable and reproducible. We analyzed RT-PCR from 20 flies in each of three independent biological replicates, and the replicates yielded consistent and comparable results. Additionally, for experiments involving HEK293T cells, we conducted three replicates and used 12-well plates with a sample size of >10000 cells per condition within each replicate, and the replicates yielded consistent and comparable results.
Randomization	F1 progenies were randomly selected from F0 crosses. We also randomly selected F1 flies for Sanger sequencing analysis.
Blinding	All fly crosses we had selection marked like white +, yellow+, or GFP in eyes to identify appropriate genotype of progenies. Investigators were not blinded with all these scoring genotypes. Investigators were not blinded with HEK293T cell line experiments since fluorescence presence was used for cell sorting.

Reporting for specific materials, systems and methods

We require information from authors about some types of materials, experimental systems and methods used in many studies. Here, indicate whether each material, system or method listed is relevant to your study. If you are not sure if a list item applies to your research, read the appropriate section before selecting a response.

Materials & experimental systems

n/a	Involved in the study	
☒	☐	Antibodies
☐	☒	Eukaryotic cell lines
☒	☐	Palaeontology and archaeology
☐	☒	Animals and other organisms
☒	☐	Clinical data
☒	☐	Dual use research of concern
☒	☐	Plants

Methods

n/a	Involved in the study	
☒	☐	ChIP-seq
☐	☒	Flow cytometry
☒	☐	MRI-based neuroimaging

Eukaryotic cell lines

Policy information about cell lines and Sex and Gender in Research

Cell line source(s)	HEK293T cells were purchased from ATCC CRL-3216.
Authentication	None of the cell lines have been authenticated
Mycoplasma contamination	Cell lines were not tested for mycoplasma contamination but no indication of contamination was observed.
Commonly misidentified lines (See ICLAC register)	No commonly misidentified cell lines were used

Animals and other research organisms

Policy information about studies involving animals; ARRIVE guidelines recommended for reporting animal research, and Sex and Gender in Research

Laboratory animals	Drosophila melanogaster strain and age of flies are reported in this study.
Wild animals	No wild animals were used in this study.
Reporting on sex	Sex and genotype of flies are reported in this study.
Field-collected samples	No field collection was carried out.
Ethics oversight	All fly crosses were carried out in glass vials in ACL-1 facility

Note that full information on the approval of the study protocol must also be provided in the manuscript.

Flow Cytometry

Plots

Confirm that:

- [x] The axis labels state the marker and fluorochrome used (e.g. CD4-FITC).
- [x] The axis scales are clearly visible. Include numbers along axes only for bottom left plot of group (a 'group' is an analysis of identical markers).
- [x] All plots are contour plots with outliers or pseudocolor plots.
- [x] A numerical value for number of cells or percentage (with statistics) is provided.

Methodology

Sample preparation	HEK293T cells were grown for 72 hrs after transfection, washed with PBS, diluted in FACS buffer (2%FBS, 2mM NaN3 in PBS) and subsequently analyzed using FACS.
Instrument	FACS aria
Software	FACSDiva (v9.0)
Cell population abundance	Sample size of >10000 cells per condition within each replicates were acquired for FACS analysis.
Gating strategy	FSC-A/FSC-H (doublet exclusion)>FSC-A/SSC-A (Cell of interest)

- [x] Tick this box to confirm that a figure exemplifying the gating strategy is provided in the Supplementary Information.
